# Noninvasive Real-Time Characterization of Renal Clearance Kinetics in Diabetic Mice after Receiving Danshensu Treatment

**DOI:** 10.1155/2018/8267560

**Published:** 2018-02-12

**Authors:** Lei Gao, Yiu-Wa Kwan, Andrew C. Bulmer, Christopher W. K. Lai

**Affiliations:** ^1^Department of Health Technology and Informatics, Hong Kong Polytechnic University, Kowloon, Hong Kong; ^2^School of Biomedical Sciences, Chinese University of Hong Kong, New Territories, Hong Kong; ^3^School of Medical Science and Menzies Health Institute Queensland, Griffith University, Gold Coast, Australia

## Abstract

Danshensu (DSS) is an active ingredient extracted from the root of the Danshen that could ameliorate oxidative stress via upregulation of heme oxygenase- (HO-) 1. Little is known about the treatment effects of DSS on kidney function in diabetic mice. Therefore, the primary aim of the present study was to characterize the renal clearance kinetics of IRdye800CW in *db/db* mice after DSS treatment. The secondary aim was to measure several biomarkers of renal function and oxidative stress (urinary F2-isoprostane, HO-1 in kidney and serum bilirubin). Fourteen *db/db* diabetic mice were randomly assigned into two groups and received either DSS treatment (DM + DSS) or vehicle treatment (DM). A third group that comprised of *db/+* nondiabetic mice (non-DM control) received no DSS treatment and served as the nondiabetic control. At the end of a 3-week intervention period, serum and urinary biomarkers of renal function and oxidative stress were assessed and the renal clearance of IRdye800CW dye in all mice was determined noninvasively using Multispectral Optoacoustic Tomography. The major finding from this study suggested that DSS treatment in *db/db* mice improved renal clearance. Increased expression of HO-1 after DSS treatment also suggested that DSS might represent a potential therapeutic avenue for clinical intervention in diabetic nephropathy.

## 1. Introduction

The hyperglycemic and hyperinsulinemic conditions in diabetes are major risk factors promoting lipid peroxidation [1–3] and impair kidney function [4–6]. Growing evidence indicates that heme oxygenase- (HO-) 1 and unconjugated bilirubin are potent antioxidants with therapeutic potential in diabetes [7–9]. Many bioactive compounds extracted from natural medicinal herbs/fruits, including Danshensu (DSS) and Paeonol, may hold beneficial antioxidant and antiapoptotic effects, mediated via activation of factor-erythroid 2-related factor 2 (Nrf2)/HO-1 signaling [10]. DSS, an active ingredient extracted from the root of the Danshen (*Salvia miltiorrhiza*), has been used for the treatment of cardiovascular disease [11, 12]. Also, the renoprotective effect of DSS has previously been linked with the suppression of oxidative stress [13], inflammation, and fibrosis [14], in addition to a reduction in lipid peroxidation by scavenging free radicals and preventing thiol oxidation [15, 16]. Moreover, the combined prescription of DSS with *Rheum rhabarbarum* is a well-recognized, effective, and safe traditional Chinese medicinal regimen for treating chronic kidney disease [17] and suppressing oxidative stress [18, 19].

Insulin glomerular filtration rate currently represents the gold standard assessment method of renal function. However, with recent advances in photoacoustic imaging, assessment of renal function in small animals (including the assessment of IRdye800CW renal clearance) can now be determined noninvasively using Multispectral Optoacoustic Tomography (MSOT). MSOT is an emerging technique that captures photoacoustic signals from chromophoric spectra or molecules that are distributed within tissues [20]. With the development of new imaging probes [21], photoacoustic imaging has now been applied to visualize the anatomy, function, and blood oxygenation in different organs [22, 23]. Yet, the assessment of DSS on renal clearance kinetics in a diabetic mice model has not been investigated to date.

Inadequate HO-1 expression has been demonstrated in obese diabetic mice [24], and the systemic induction of HO-1 can improve insulin sensitivity, decrease inflammatory cytokine expression, and increase circulating adiponectin [25, 26]. Also, the induction of HO-1 within renal structures normalized blood pressure, protected against oxidative injury, and consequently improved renal function in spontaneously hypertensive rats [27]. Bilirubin is generally considered as the by-product of heme catabolism. However, new evidence suggests that it may also possess physiological significance. Despite the uncertainty of its physiological importance, unconjugated bilirubin has demonstrated potent antioxidant capacity in vitro and ex vivo [28, 29]. An argument for a physiological role of bilirubin is further supported by reduced bilirubin concentrations in patients who had chronic kidney disease [30]. Similarly, individuals with elevated serum bilirubin have decreased prevalence of kidney complications in diabetes [9]. These findings, therefore, support that HO-1 and bilirubin might protect the kidney from oxidative stress by acting as an antioxidant [31–33]. The abrogation of Nrf2/HO-1-dependent signaling cascade has been largely implicated in chronic/acute kidney injury, cardiac/endothelial dysfunction, and cerebral ischemia [34]. Many researchers have demonstrated that DSS-mediated tissue protection against chronic kidney disease occurs via cytoprotective and prosurvival Nrf2/HO-1 and PI3K/Akt signaling pathways [10, 35]. However, whether overexpression of HO-1 is implicated in the DSS treatment effect in diabetic renal function remains unknown. In this regard, the present study aimed to (1) characterize the renal clearance kinetics of IRdye800CW dye in *db/db* mice after DSS treatment and (2) quantify the expression of several biomarkers for renal function and oxidative stress in *db/db* mice with and without DSS treatment.

## 2. Materials and Methods

### 2.1. Animals and Intervention

Female 10 wk old diabetic homozygous (*db/db*) mice and nondiabetic heterozygous (*db/+)* mice on a C57BLKS/J background were housed in the Central Animal facilities, Hong Kong Polytechnic University, in a 12 h light/dark cycle and under tight control of temperature and humidity. The *db/db* homozygotes exhibit persistent hyperphagia and obesity with spontaneously developed elevated leptin, glucose, and insulin concentrations [36]. All mice received regular laboratory chow and tap water ad libitum during the study. After 1 week of acclimation, all diabetic mice were randomly divided into two groups (*n* = 7/group): DM and DM + DSS, while heterozygote nondiabetic mice (*n* = 6) were assigned to a non-DM control group. During the intervention period of 3 weeks, all mice were treated according to the following schedule: the non-DM control group received no treatment, the DM group received i.p. vehicle treatment while the DM + DSS group received DSS (HPLC ≥ 98%, dissolved in water, Nanjing Zelang Pharmaceutical Technology Co. Ltd.) at a dose of 10 mg/kg i.p. daily. The kidney absorption level of DSS was found to be at around 69 *μ*g/g of tissue via i.p. method [37]. Experimental protocols were performed in accordance with the approved license granted under the Department of Health and approved by the Animal Subjects Ethics Sub-Committee (ASESC) of Hong Kong Polytechnic University.

### 2.2. Fasting Glucose, Body Weight, and Urinary Samples

At the start and the end of the study, fasting blood glucose was assessed using a glucometer (Bayer Contour TS), and the body weight of mice was assessed using an electronic scale. Daily urinary samples were collected, for four days before the end of the study using individual metabolic cages for the determination of F2-isoprostane (IsoPs), microalbumin, and creatinine excretion of each mouse. The 24 hr urinary concentration of IsoPs was determined by commercial ELISA method (item number 516351, Cayman Chemical, Ann Arbor, Michigan, USA), while the levels of urinary albumin and creatinine were determined using a clinical chemical analyzer (AU480; Beckman Coulter, Brea, CA, USA).

### 2.3. Serum and Kidney Samples

After overnight fasting, the mice were sacrificed and blood samples were collected via cardiac puncture at the end of the study. Serum concentrations of creatinine and bilirubin (total, conjugated, and unconjugated) were assessed using clinical chemistry (AU480; Beckman Coulter, Brea, CA, USA). The concentration of fasting serum insulin was assayed by commercial ELISA method (catalogue number 32270; Li Ka Shing Faculty of Medicine, the University of Hong Kong, Hong Kong). The cortex of the kidney was carefully dissected for the analysis of HO-1, p-Akt, and t-Akt expression using western blot. The total protein concentration was determined using a Bio-Rad Protein Assay Kit II (Bio-rad, catalog number 500-0002). The blots were incubated with primary antibodies overnight, including HO-1 Antibody (Cell Signaling Technology, Beverly, MA, USA), pan-Akt (Cell Signaling Technology, Beverly, MA, USA), and Phospho-Akt^Thr308^ (Cell Signaling Technology, Beverly, MA, USA). After washing, blots were incubated with horseradish peroxidase- (HRP-) conjugated secondary antibody (Santa Cruz Biotechnology). Finally, protein expression was determined by a microplate reader (Bio-Rad Laboratories, Richmond, CA) and quantified using ImageJ software (IJ 1.46r).

### 2.4. Measurement of Renal Clearance of IRdye800CW Dye Using MSOT

**Figure 1:**
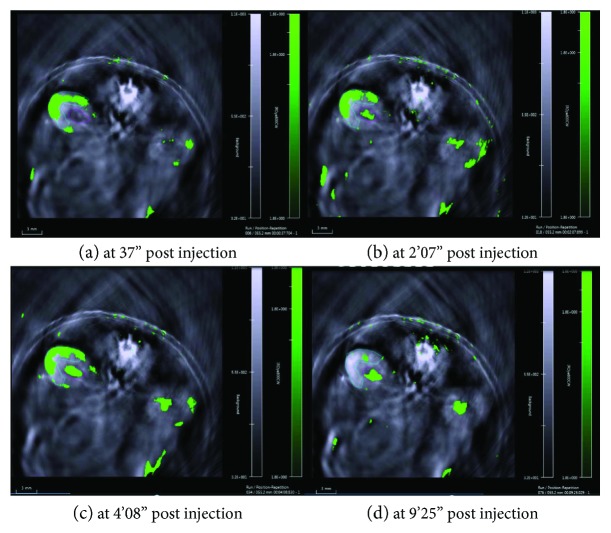
The transition of IRdye800CW peak signal intensity (from the renal cortex and renal pelvis) in the right kidney over time in a *db/db* mouse with no DSS treatment.

On the last day of the intervention period, all mice were anesthetized using isoflurane in oxygen [3-4% per liter of 100% oxygen for induction and 1.5% per liter of 100% oxygen during maintenance], with hair removed from the chest to lower abdomen as per previously published experimental protocols [38]. In brief, mice were put into a water chamber within the MSOT (inVision 128 MSOT imaging system, iThera Medical, Munich, Germany) in a prone position, and the kidney region was then scanned at a rate of 10 Hz continuously using a multispectral protocol for 10 minutes after injection of 200 *μ*l (20 nmol in 0.9% of saline) of IRdye800CW (LI-COR, USA) via the tail vein (Figure 1). IRdye800CW is a small molecule that is rapidly excreted by the kidneys in unmetabolised form [39]. After multispectral decomposition of IRdye800CW signals over the anatomical background, the time points at the mean of peak signal intensity (Tmax) over the renal cortex and renal pelvis regions of the right kidney were determined, and the time difference between Tmax-Pelvis and Tmax-Cortex was calculated as “Tmax delay,” which represents the efficiency of IRdye200CW dye clearance [38].

### 2.5. Statistical Analyses

The assumptions of normality and homogeneity of variance were first assessed. ANOVA with multiple post hoc LSD adjustments or Kruskal-Wallis H test with multiple post hoc Dunn adjustments was used to compare the differences in the three groups where applicable. Paired *t*-tests were used to test for significant differences between the start and end fasting glucose concentrations in each group. All data were expressed as means ± SD. All statistical analyses were performed using the Statistical Package for the Social Sciences (SPSS) version 22 for Windows, and the significant level was set at *p* < 0.05.

## 3. Results

**Figure 2:**
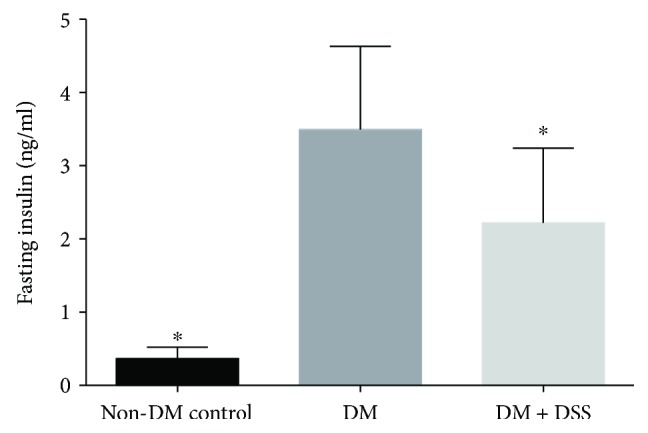
The fasting insulin level of mice at the end of the 3-week intervention period (*n* = 6 − 7/group). ∗ denotes *p* < 0.05 when compared to the DM group.

**Figure 3:**
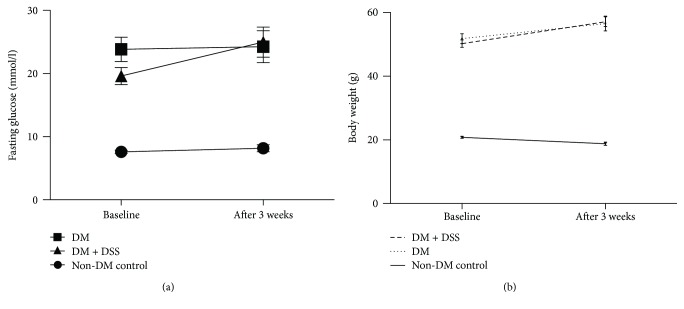
Changes in fasting blood glucose concentration (a) and body weight (b) of mice before and after 3 weeks of intervention.

A summary of all measured variables collected from serum, urine, and MSOT in the present study can be found in Table 1. The results indicated that all *db/db* mice exhibited hyperglycemia and hyperinsulinemia and were more obese (Figure 2) when compared to *db/+* mice at baseline and after 3 weeks. However, the fasting insulin concentration at the end of the study in the DM group (3.50 ± 1.14 nmol/l) was significantly greater when compared to those in the DM + DSS (2.22 ± 1.02 nmol/l, *p* = 0.035) and non-DM control (0.37 ± 0.16 nmol/l, *p* = 0.007) groups, suggesting that DSS treatment might improve insulin resistance in the *db/db* mice. On the contrary, there was no significant change in fasting glucose and body weight between baseline and after 3 weeks in all groups, except that the fasting blood glucose concentration tends to be increased in the DM + DSS group (from 19.60 ± 1.35 mmol/l at baseline to 24.97 ± 2.39 mmol/l after 3 weeks, *p* = 0.098) (Figure 3).

### 3.1. DSS Treatment Failed to Reduce ACR and Serum Creatinine Level but Improved the Tmax Delay (Renal Clearance) in *db/db* Mice

**Figure 4:**
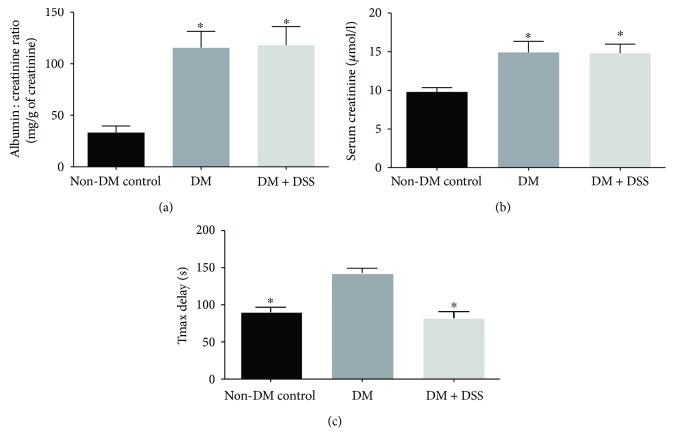
Kidney function markers: urinary albumin : creatinine ratio (a), serum creatinine (b), and Tmax delay (s) of mice after 3 weeks of intervention. ∗ denotes *p* < 0.05 when compared to the non-DM control group in (a) and (b) and when compared to the DM group in (c).

**Figure 5:**
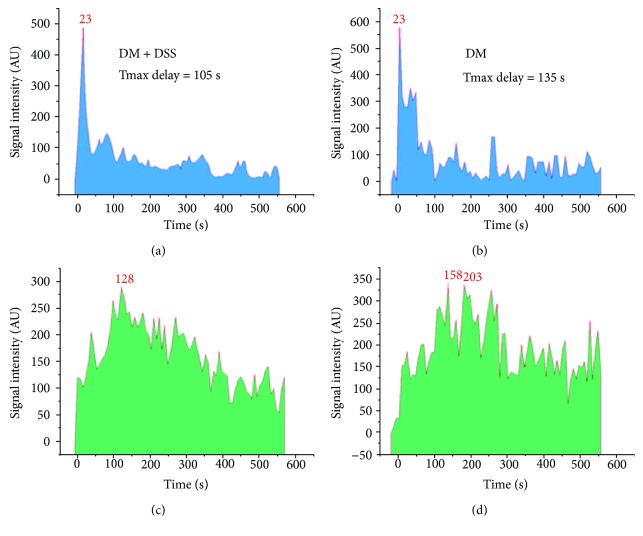
A plot of signal intensity over time over the renal cortex and renal pelvis of the kidney. (a, c) The left shows the spectrum collected from a mouse in the DM + DSS group [peak at 23 s and 128 s in the renal cortex (a) and renal pelvis (c), respectively; Tmax delay = 105 s]. (b, d) The right shows the spectra collected from a mouse in the DM group [peak at 23 s and 158 s in the renal cortex (b) and renal pelvis (d), respectively; Tmax delay = 135 s].

Both the DM and DM + DSS groups demonstrated increased urinary albumin : creatinine ratio (ACR) (Figure 4(a)) and serum creatinine (Figure 4(b)) when compared to the non-DM control group, which was consistent with a previous study [40]. From the graphs shown in Figure 5, Tmax delay determined by MSOT was longer in a *db/db* mouse without DSS treatment (Figures 5(a) and 5(c)) when compared to another *db/db* mouse with DSS treatment (Figures 5(b) and 5(d)). Collectively, the mean value of Tmax delay was significantly longer in the DM group when compared to the DM + DSS (*p* = 0.001) and non-DM control (*p* < 0.001; Figure 4(c)) groups, suggesting an improved renal clearance after DSS treatment in the DM + DSS group.

### 3.2. DSS Treatment Did Not Increase Serum Bilirubin or Significantly Reduce Urinary F2-Isoprostane Concentrations in *db/db* Mice

**Figure 6:**
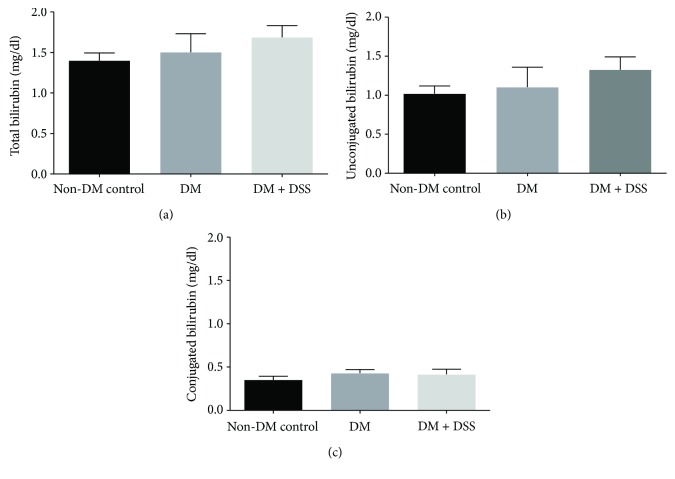
The serum total bilirubin (a), unconjugated bilirubin (b), and conjugated bilirubin (c) levels of mice after 3 weeks of intervention period (*n* = 6 − 7/group).

**Figure 7:**
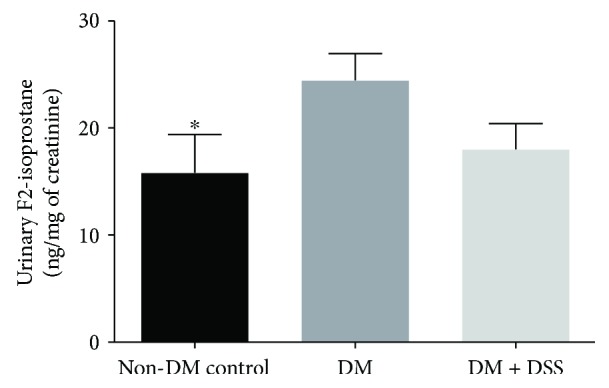
The urinary F2-isoprostane concentration of mice at after 3 weeks of intervention. ∗ denotes *p* < 0.05 when compared to the DM group.

In the present study, the total bilirubin, unconjugated bilirubin, and conjugated bilirubin levels in the three groups (Figure 6) were similar, and the result was comparable to a previous reported study [41]. Although the DM + DSS group exhibited the highest concentrations of total bilirubin and unconjugated bilirubin when compared to the non-DM and DM groups, this difference did not reach statistical significance (all *p* values > 0.2). On the other hand, after the 3-week intervention period, the DM (24.46 ± 2.49 ng/mg) group demonstrated a greater urinary concentration of F2-isoprostane when compared to the non-DM control (18.01 ± 2.41 ng/mg, *p* = 0.046) group. With DSS treatment, urinary F2-isoprostane over 24 hours reduced to 15.81 ± 3.56 ng/mg in the DM + DSS group, although this reduction was not statistically significant (*p* = 0.113) when compared to the DM group (Figure 7).

### 3.3. Upregulation of HO-1 Expression in the Kidney of Diabetic Mice after 3 Weeks of DSS Treatment

**Figure 8:**
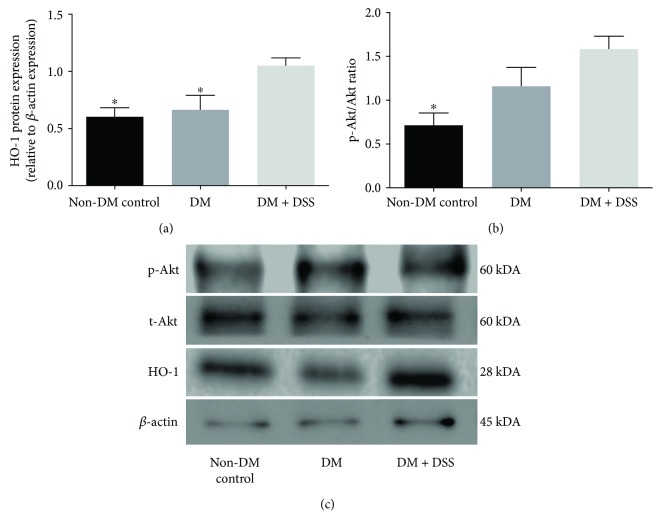
The expression of HO-1 (a) and p-AKT/t-Akt ratio (b) of mice at the end of the 3-week intervention period. *N* = 4 in each group, and the corresponding western blot data of HO-1 and Akt (c). ∗ denotes *p* < 0.05 when compared to the DM + DSS group.

Finally, we analyzed the renal cortex for expression of HO-1 and the p-Akt/t-Akt ratio. Significantly increased expression of HO-1 (~2-fold) was noted in the DM + DSS group when compared to the DM (*p* = 0.029) and non-DM control (*p* = 0.016) groups (Figure 8(a)). Although the p-Akt/t-Akt ratio was also significantly increased (~3-fold, *p* = 0.011) in the DM + DSS group when compared to the non-DM group, the mean difference of the p-Akt/t-Akt ratio between the DM + DSS and DM groups remained insignificant (*p* = 0.125; Figure 8(b)). The corresponding western blot data of HO-1 and AKT are presented in Figure 8(c).

## 4. Discussion

### 4.1. DSS Treatment and Diabetic Status

*db/db* mice spontaneously develop hyperinsulinemia due to mutation in the leptin receptor, which leads to impaired function of beta cells of the pancreatic islets. At 4 weeks of age, hyperglycemia, hyperinsulinemia, and insulin resistance are observed [42]. In the present study, although there was no observable change in the fasting glucose level in diabetic mice after DSS treatment, fasting insulin concentrations in the DSS treatment group was decreased when compared to nontreated diabetic group. This finding agreed with a previously published study [13], suggesting the possibility of improved insulin sensitivity mediated by DSS.

### 4.2. DSS Treatment and Renal Clearance

Significant reduction in renal function was evidenced in diabetic mice of the present study, as indicated by higher ACR and serum creatinine when compared to the nondiabetic group. However, the DSS antioxidant treatment failed to ameliorate the serum creatinine level, probably due to the difference in the injection approach and hence a lower daily effective dosage of DSS employed in the present study when compared to other published studies [43, 44]. ACR and serum creatinine are conventional and clinically relevant parameters for the assessment of kidney function and are significantly correlated with oxidative stress due to inactivation of NO [45]. However, proteinuria and changes in circulating creatinine concentrations or clearance have their limitations in regard to sensitivity and are typically modulated in moderate and late stages of renal disease. Therefore, we applied a novel, noninvasive measurement of renal clearance kinetics to determine the impact of DSS on renal function in diabetic animals, using the same methodology suggested by Scarfe's group [38]. This noninvasive examination technique provides a clear, sensitive, and specific optical signal from the target tissue with the utilization of IRDye800CW. Our results of Tmax delay in our diabetic mouse model were similar to the previous work that studied the acute effect of adriamycin-induced nephropathy on Tmax delay [38]. However, our results on Tmax delay have the following limitations. Firstly, it should be noted that Tmax delay mainly assesses the hyperfiltration of IRdye800CW and does not account for tubular reabsorption of metabolites in the kidney and variations in hourly production of creatinine. Second, IRdye800CW could bind to plasma proteins and lead to underestimation of the true “Tmax delay” in the present study [38].

### 4.3. DSS Treatment and Lipid Peroxidation

DSS treatment was previously reported to ameliorate oxidative stress and lipid peroxidation via Akt/Nrf2/HO-1 [46, 47]. Lipid peroxidation is elevated in patients with diabetes, especially in those with increased HbA1c, LDL cholesterol, total cholesterol, and triglycerides [48]. In obese and diabetic patients, the accumulation of lipids and advanced glycation end products in plasma or organs represents an important source of lipid peroxidation, which further leads to DNA damage, protein/enzyme oxidation, and release of proinflammatory cytokines [49–51]. Many previous studies have shown that urinary IsoPs are a reliable biomarker of lipid peroxidation and could act as an indicator of oxidative stress [52, 53]. In the present study, diabetic mice exhibited higher levels of urinary IsoPs when compared to nondiabetic controls, which agrees with previous findings [54, 55]. However, the 3-week period of DSS treatment failed to significantly reduce the urinary IsoP concentration in *db/db* mice. At present, only a few studies have investigated the effect of *Salvia miltiorrhiza* (containing DSS) treatment on IsoPs [56, 57], with most results indicating that DSS-containing herbs could attenuate IsoPs in nondiabetic murine models. Therefore, our study is the first report to investigate the effect of DSS specifically on IsoP in *db/db* mice.

### 4.4. DSS Treatment and HO-1 Expression

We postulated that DSS is a potential druggable adjuvant in ameliorating diabetic nephropathy via induction of HO-1 synthesis. Previous studies have indicated that the HO system may act as a crucial mediator of cellular redox homeostasis by degrading heme, generating the antioxidant bilirubin, and releasing free iron (bound by ferritin) especially in the renovascular system [27, 58, 59]. Through activation of the nuclear factor-erythroid 2-related factor-2- (Nrf2-) targeting antioxidant response element (*ARE*)/heme oxygenase-1 (HO-1) signaling cascade, DSS has attenuated acute kidney injury [35]. The induction of HO-1 further activated adiponectin synthesis/release, which in turn improved cellular redox status, diminished apoptotic signaling kinase-1 expression, and protected from oxidative stress via activating p-Akt/Akt signaling [59–61]. In the present study, although DSS treatment was associated with increased expression of HO-1 in the kidney of *db/db* mice when compared to DSS-treated *db/db* mice, the levels of total and unconjugated bilirubin in the blood were only mildly elevated, suggesting an argument against HO-1-mediated protection via bilirubin in our diabetic mice model. According to a previous study, downregulation of Akt could attenuate the antioxidant effects of HO-1 [62]; however, our data demonstrate that DSS could only mildly elevate the p-Akt : t-Akt ratio. Therefore, the failure of DSS-induced overexpression of bilirubin and Akt suggests other key players might be involved in mediating the beneficial effects of HO-1, such as carbon monoxide (CO) production or improved heme clearance. In this context, further studies on the effect of DSS treatment on CO production and heme clearance are warranted.

This study has several limitations. First, our team failed to collect enough blood for the baseline measurement of all selected biomarkers in the present study. Therefore, we only measured the serum fasting glucose at baseline, which required minimal blood volume. Second, the tail vein cannot be recovered within 3 weeks after the injection of dye; therefore, we could not complete the baseline measurement of renal clearance.

In summary, this study suggests that DSS might represent a potential viable preventative/treatment worthy of further investigation in patients with, or at risk of developing, diabetic nephropathy. Although HO-1 is known to ameliorate diabetic nephropathy [63], its effect in *db/db* mice remained poorly understood. In the present study, DSS treatment significantly improved renal clearance in *db/db* mice and was associated with upregulation of HO-1/Akt signaling pathways. However, the exact mechanism concerning how DSS mediates HO-1 activity and preserves renal physiological function remains unknown and requires further study.

## Figures and Tables

**Table 1:** A summary of all measured variables collected from serum, urine, and MSOT in the present study.

		Non-DM control	DM	DM ± DSS
*Baseline measurement*				
Obesity	Body weight (g)	20.8 ± 0.69	51.79 ± 4.03^∗^	50.21 ± 3.08^∗^
Serum	Fasting glucose (mmol/l)	7.58 ± 0.60	23.83 ± 5.10^∗^	19.60 ± 3.58^∗^
*After 3 weeks*				
Obesity	Body weight (g)	18.78 ± 1.21	56.52 ± 6.07^∗^	57.09 ± 4.03^∗^
Serum	Fasting glucose (mmol/l)	8.17 ± 1.36	24.24 ± 6.67^∗^	24.97 ± 6.32^∗^
	Creatinine (*μ*mol/l)	9.79 ± 1.37	14.90 ± 3.79^∗^	14.80 ± 3.10^∗^
	Total bilirubin (*μ*mol/l)	1.40 ± 0.21	1.50 ± 0.51	1.69 ± 0.32
	Conjugated bilirubin (*μ*mol/l)	0.35 ± 0.10	0.43 ± 0.11	0.41 ± 0.16
	Unconjugated bilirubin (*μ*mol/l)	1.02 ± 0.23	1.10 ± 0.58	1.33 ± 0.37
Urine	F2-isoprostane (ng/mg of creatinine)	15.82 ± 8.73^#^	24.46 ± 6.59	18.00 ± 6.39
	Urinary albumin : creatinine ratio (mg/g of creatinine)	33.5 ± 14.86	115.7 ± 41.54^∗^	118.2 ± 47.31^∗^
MSOT	Tmax delay (s)	90.21 ± 14.93	142.97 ± 13.96^∗^	81.96 ± 19.51

All data presented as mean ± SD. ∗ denotes *p* < 0.05 when this group is compared to the non-DM control group. # denotes *p* < 0.05 when this group is compared to the DM group.

## Supplementary Materials

Supplementary Materials A video showing the signal intensity of IRdye800CW accumulated in the kidneys of a mouse after postinjection of IRdye800CW for 15 minutes. The region of interest in blue represents the renal cortex of the right kidney while the region of interest in purple represents the renal pelvis. The strength (in arbitrary units) of the photoacoustic signals from both the anatomical background and IRdye800CW dyes were denoted as different grey scale and green color on the right side of the panel.
